# Author Correction: De novo assembly, characterization, functional annotation and expression patterns of the black tiger shrimp (*Penaeus monodon*) transcriptome

**DOI:** 10.1038/s41598-022-11096-w

**Published:** 2022-05-04

**Authors:** Roger Huerlimann, Nicholas M. Wade, Lavinia Gordon, Juan D. Montenegro, Jake Goodall, Sean McWilliam, Matthew Tinning, Kirby Siemering, Erika Giardina, Dallas Donovan, Melony J. Sellars, Jeff A. Cowley, Kelly Condon, Greg J. Coman, Mehar S. Khatkar, Herman W. Raadsma, Gregory E. Maes, Kyall R. Zenger, Dean R. Jerry

**Affiliations:** 1 ARC Research Hub for Advanced Prawn Breeding, Townsville, QLD 4811 Australia; 2 Centre for Sustainable Tropical Fisheries and Aquaculture, College of Science and Engineering, James Cook University, Townsville, QLD 4811 Australia; 3 Aquaculture, CSIRO Agriculture and Food, 306 Carmody Road, St Lucia, QLD 4067 Australia; 4 Australian Genome Research Facility Ltd, The Walter and Eliza Hall Institute, 1G Royal Parade, Parkville, VIC 3050 Australia; 5 Seafarms Group Ltd, Level 11 225 St Georges Terrace, Perth, WA 6000 Australia; 6 Sydney School of Veterinary Science, Faculty of Science, The University of Sydney, Sydney, NSW Australia; 7 Aquaculture, CSIRO Agriculture and Food, 144 North Street, Woorim, QLD 4507 Australia; 8 Laboratory of Biodiversity and Evolutionary Genomics, KU Leuven, 3000 Leuven, Belgium; 9 Center for Human Genetics, UZ Leuven- Genomics Core, KU Leuven, 3000 Leuven, Belgium

Correction to: *Scientific Reports* https://doi.org/10.1038/s41598-018-31148-4, published online 10 September 2018

The original version of this Article contained errors in the Results section under subheading ‘Virus discovery’ where,

“The remaining 21 contigs had Top Hit E-value scores identifying them to be related most closely to strains of Gill-associated virus (GAV; 4 contigs, longest 26,235 nt), *Penaeus chinensis* hepandenovirus (*Pchi*HDV; 4 contigs, longest 1,884 nt), Wenzhou shrimp virus 2 (WSV2; RdRp, hypothetical protein and G protein contigs, longest 6,891 nt), Wenzhou shrimp virus 8 (WSV8; 6 contigs, longest 4,579 nt), Beihai picorna-like virus 2 (5,277 nt), Wenzhou picorna-like virus 23 (551 nt) and Moloney murine leukaemia virus Pr180 sequence (Mo-MuLV; 2,431 nt).”

now reads:

“The remaining 21 contigs had Top Hit E-value scores identifying them to be related most closely to strains of Gill-associated virus (GAV; 4 contigs, longest 26,235 nt), *Penaeus chinensis* hepandenovirus (*Pchi*HDV; 4 contigs, longest 1,884 nt), Whenzhou shrimp virus 2 (When-2; RdRp, hypothetical protein and G protein contigs, longest 6,891 nt), Whenzhou shrimp virus 8 (When-8; 6 contigs, longest 4,579 nt), Beihai picorna-like virus 2 (5,277 nt), Wenzhou picorna-like virus 23 (551 nt) and Moloney murine leukaemia virus Pr180 sequence (Mo-MuLV; 2,431 nt).”

In the Discussion section,

“The presence of near full-length ssRNA genome sequences for viruses such as gill-associated virus (GAV, 26,235 nt) and white spot virus 2 (WSV2, 10,542 nt) provided additional validation of the methods used to synthesize and assemble the transcriptome, and to its completeness as demonstrated by various metrics measuring the nature and number of endogenous gene transcripts.”

now reads:

“The presence of near full-length ssRNA genome sequences for viruses such as gill-associated virus (GAV, 26,235 nt) and two sequences (deposited on NCBI: OM219076 and OM219077, cumulative length of 10,542 nt ) with high similarity to Whenzhou shrimp virus-2 L and M segments (When-2, KM817720.2 and KM817687.1) provided additional validation of the methods used to synthesize and assemble the transcriptome, and to its completeness as demonstrated by various metrics measuring the nature and number of endogenous gene transcripts.”

“In addition to known endemic viruses, the transcriptome contained full-length or near full-length RNA transcripts related closely to the recently-described shrimp viruses WSV2 and WSV8^50,51^ unknown until now to occur in Australian *P*. *monodon*.”

now reads:

“In addition to known endemic viruses, the transcriptome contained full-length or near full-length RNA transcripts related closely to the recently-described shrimp viruses When-2 and When-8^50,51^ unknown until now to occur in Australian *P*. *monodon*.”

The original Article has been corrected.

